# Study on the mechanism of andrographolide activation

**DOI:** 10.3389/fnins.2022.977376

**Published:** 2022-09-13

**Authors:** Qihan Cai, Weina Zhang, Yanan Sun, Lu Xu, Mengmeng Wang, Xinliang Wang, Siming Wang, Zhiyu Ni

**Affiliations:** ^1^School of Basic Medical Science, Hebei University, Baoding, China; ^2^Hebei Institute of Dermatology, Baoding, China; ^3^College of Traditional Chinese Medicine, Hebei University, Baoding, China; ^4^Affiliated Hospital of Hebei University, Baoding, China; ^5^Clinical Medical College, Hebei University, Baoding, China; ^6^Hebei Collaborative Innovation Center of Tumor Microecological Metabolism Regulation, Baoding, China

**Keywords:** andrographolide, anti-inflammatory, anti-tumor, antiviral, nervous system, NF-κB, AKT

## Abstract

Andrographolide is a natural antibiotic that has the ability to dispel heat, detoxify, reduce inflammation, and relieve pain. Recent research has shown that it can exert anti-inflammatory effects *via* multiple pathways and multiple targets (mediated by NF-κB, JAK/STAT, T cell receptor, and other signaling pathways). It can inhibit human lung cancer cells, colon cancer cells, osteosarcoma cells, and other tumor cells, as well as reduce bacterial virulence and inhibit virus-induced cell apoptosis. It can also regulate inflammatory mediator expression to protect the nervous system and effectively prevent mental illness. Additionally, andrographolide regulates the immune system, treats cardiovascular and cerebral vascular diseases, protects the liver, and the gallbladder. It is clear that andrographolide has a huge range of potential applications. The mechanism of andrographolide’s anti-inflammatory, antibacterial, antiviral, and nervous system defense in recent years have been reviewed in this article.

## Introduction

*Andrographis* is a member of the family andrographolide. It is cold in nature and bitter in taste. It has the properties of clearing heat and detoxifying, as well as cooling the blood and reducing swelling. *Andrographis* is widely cultivated in tropical and subtropical areas, and it have its origins in South India and Sri Lanka (Lee et al., 2010; Jiao et al., 2019). In the 1950s, it was imported into China from Southeast Asia and was primarily produced in the provinces of Guangxi, Guangdong, Sichuan, Anhui, Fujian, and others (Zhang X. et al., 2018).

The main components of *Andrographis* are andrographolide, neoandrographolide, 14-deoxy-11, 12-dehydroandrographolide (14-DDA), 14-deoxy-andrographolide (14-DA), anthocyanins, anthocyanins A, B, C (Jin et al., 2012; Aromdee, 2014), with anti-inflammatory, antiviral, antibacterial, and anti-cancer properties, liver and gallbladder protection, as well as some other pharmacological effects (Jin et al., 2012; Lim et al., 2012). *Andrographis* has been widely used to treat sore throats, flu, and upper respiratory infections in Asian countries such as China, India, Thailand, and Malaysia since the last century (Ghosh et al., 2011). Some studies have found that the application of products from traditional plants (such as *Moringa oleifera* and *Psoralea corylifolia*) has important significance for some effective new molecules [such as 4-(α-l-rhamnosyloxy)-benzyl isothiocyanate and Bakuchiol] in anti-tuberculosis treatment, and andrographolide isolated from *Andrographis* has excellent anti-mycobacterium activity and predicted the potential target of andrographolide in *Mycobacterium tuberculosis* (Gautam et al., 2007; Prabu et al., 2015). It also inhibits atherosclerosis by restraining the release of pro-inflammatory cytokines such as MCP-1 and IL-6, reactive oxygen species (ROS) production and foam cell formation (Wu et al., 2018). Andrographolide could also be used in mental illness treatment. Previous studies indicated that andrographolide improved motor and neurobehavioral function in Parkinson’s disease mice by inducing mitochondrial autophagy and alleviating neuroinflammation, the effect is dose-dependent (Geng, 2016; Wang et al., 2022). Andrographolide enhanced hippocampal neurogenesis by increasing brain-derived neurotrophic factor (BDNF) expression in mice, as well as having antidepressant and anti-inflammatory effects in chronic unpredictable mild stress (CUMS)-induced mice (Geng et al., 2019; Zhang et al., 2019). Overall andrographolide is a diterpenoid lactone which has potential for synovitis, mastitis, depression, Parkinson’s disease as well as immune function improvement (Geng, 2016; Li et al., 2017; Wan, 2017; Geng et al., 2019; Lin and Xu, 2019; Ren, 2019; Wang et al., 2022). This review elaborates on the mechanism of action of andrographolide.

## Anti-inflammatory mechanism

Cytokine is a small-molecule polypeptide or glycoprotein that immune cells and some non-immune cells synthesize and secrete. Cytokines can effectively mediate cell interactions and perform a variety of biological functions. They play an important role in the occurrence and maintenance of inflammatory diseases, and inhibition of inflammatory mediators is an effective way to treat inflammatory diseases (Gong, 2004). Previous studies have found that andrographolide can exert anti-inflammatory effects through multiple pathways and multiple targets, including regulation of the synthesis and secretion of inflammatory mediators such as tumor necrosis factor-α (TNF-α) and interleukin. By regulating the expression and activation of nuclear transcription factor-κB (NF-κB), Toll-like receptor 4 (TLR-4), silencing information regulator 1 (SIRT1)/extracellular regulatory kinase (ERK), and other signaling pathways. Andrographolide can reduce the amount of inflammatory swelling in the paws of experimental mice by blocking the JAK2/STAT3 signaling pathway and inhibiting the release of pro-inflammatory cytokines and mediators (Gupta et al., 2020a; Huan et al., 2020). As a result, we can investigate andrographolide further using these mechanisms in order to develop safe and effective anti-inflammatory drugs.

### Anti-inflammatory effects *via* the NF-κB-mediated signaling pathway

Nuclear factor-κB (NF-κB) is an important intracellular nuclear transcription factor, and many complicated diseases are closely related to the NF-κB-mediated signaling pathway. It can affect the body’s inflammatory response, immune response, apoptosis regulation, stress response, and other processes. Cytokines, microbial components, ROS, and other mediators can activate it (Liu et al., 2017). NF-κB is known to be present in almost all animal cell types, and years of research have shown that improper regulation of NF-κB is linked to inflammation, autoimmune diseases, and cancer. It regulates the abnormal expression of pro-inflammatory genes involved in many inflammatory diseases, including lung injury (Dagher et al., 2007). However, it is important to note that excessive activation of the NF-κB signaling pathway also contributes to the development of many diseases. NF-κB signaling is activated by a variety of stimuli, including pathogens, inflammatory cytokines, growth factors, ultraviolet light, and oxidative stress (Pahl, 1999). Activation of these different pathways is mediated by a number of membrane receptors, which subsequently activate various signaling pathways leading to NF-κB activation, such as Toll-like receptor (TLR), tumor necrosis factor receptor (TNFR), interleukin-1 receptor (IL-1R), T cell receptor (TCR), B cell receptor (BCR), etc. In a series of studies, small-molecule inhibitors of the NF-κB signaling pathway have been shown to have significant therapeutic potential in inflammatory diseases and cancers, with implications for future research. Cytokines produced by the NF-κB pathway can reactivate NF-κB through a positive feedback loop, further exacerbating the injury (Liu et al., 2017). In summary, when these extracellular stimuli activate the NF-κB signaling pathway *via* receptors, signaling activates the IκB kinase complex (IKK), resulting in the degradation of the IκB protein after phosphorylation and ubiquitination, as well as the release and activation of NF-κB and its transfer to specific binding sites in the nucleus and on the target gene (κB site). As a result, the transcription of target genes such as growth factors, transcription factors, and chemokines is regulated.

Andrographolide is one of a growing number of biological and biochemical NF-κB inhibitors that have been found to have anti-inflammatory effects by either blocking the signal transduction pathway that leads to NF-κB activation or inhibiting NF-κB binding activity to target genes. Andrographolide has been identified as an NF-κB inhibitor, which inhibits the NF-κB pathway. Experiments have revealed that andrographolide has a potentially valuable therapeutic value in the treatment of asthma. Andrographolide blocks the phosphorylation of inhibitory κB kinase induced by TNF-α. It plays a positive role by inhibiting the NF-κB pathway at the level of inhibitory κB kinase β activation (Bao et al., 2009). Other studies have demonstrated the positive efficacy of andrographolide, for example, andrographolide can down-regulate Mir-21-5p by targeting NF-κB, promote the expression of the PDCD4 target gene, and further inhibit the growth and metastasis of metastatic luminal breast cancer (Li, 2021). In addition to cancer, the active component of andrographolide can inhibit the lung injury induced by PM2.5 by reducing pulmonary edema, alveolar wall thickening, alveolar bleeding, inflammatory cell infiltration, and inflammatory cytokine release. The underlying mechanism is also associated with inhibition of the NF-κB pathway (Yao et al., 2021).

Some researchers, to reveal the curative effect of andrographolide derivatives, studied how andrographolide sulfonates (the main components of the xi phlogistic flat) for chronic colitis affect specific pathways, tested the 2,4,6-trinitrobenzene sulfonic acid (TNBS)-induced chronic colitis model, and discovered that the NF-κB signaling pathway is also activated by chance, and the results demonstrated that the level of P-P65 decreased and the activation of P38 and ERK1/2 in the colon tissue was inhibited after the intervention of andrographolide sulfonates (Gao et al., 2020). In addition to andrographolide sulfonates, other andrographolide derivatives also have anti-inflammatory effects on colitis. Guo et al. (2019) found that andrographolide derivative 3B significantly reduced serum levels of pro-inflammatory cytokines such as IFN-γ, IL-6, TNF-α, and IP-10 in a model of acute colitis induced by sodium dextran sulfate (DSS) and inhibited the level of P-P65, demonstrating a positive effect. Other studies have shown that andrographolide can inhibit pathological damage in the lung and immune dysfunction in Pneumonia rats infected with *Klebsiella pneumoniae*, which has been linked to the inhibition of the TLR-4/NF-κB signaling pathway (Kang et al., 2020). The intervention of andrographolide in psychiatric diseases also has promising research prospects. It has long been found that NF-κB is expressed in many nerve cells, including neurons and glial cells. NF-κB is among the most essential transcription factors in the brain (Baeuerle and Baltimore, 1996; O’Neill and Kaltschmidt, 1997; Mattson and Camandola, 2001). In recent years, andrographolide treatment has been shown to improve depression-like behavior in mice by decreasing the overexpression of pro-inflammatory mediators and cytokines (NO, COX-2, iNOS, IL-1β, IL-6, and TNF-α) and inhibiting the signaling of NF-κB (P-P65) (Zhang et al., 2019). It is worthwhile to anticipate the clinical application of andrographolide in mental diseases.

In addition, the NF-κB signaling pathway also regulates the inflammatory responses of cytokines such as interleukin 1 (IL-1), interleukin 6 (IL-6), and TNF-α, resulting in immune, inflammatory, and stress responses (Baeuerle and Henkel, 2003). TLR-4 is an innate immune recognition receptor found on cell membranes. Activation of TLR-4 directs extracellular signals to intracellular pathways, activating IL-1 receptor-associated kinase 1 (IRAK-1) and ubiquitin-protein ligase TRAF-6, and further activating NF-κB inducible enzymes. Activated NF-κB is transferred from the cytoplasm to the nucleus, where it activates relevant target genes to initiate transcription and eventually releases inflammatory factors such as TNF-α and IL-6 downstream of the pathway through this series of signaling. Zhang L. et al. (2018) demonstrated that andrographolide can reduce nucleus pulposus degradation induced by the pro-inflammatory cytokine IL-1β in patients with intervertebral disc degeneration *via* a TLR-4/MyD88/NF-κB-mediated signaling pathway, resulting in an anti-inflammatory effect. It has been suggested that andrographolide may exert anti-inflammatory properties by inhibiting the activation of P38 MAPKs and the expression of inducible nitric oxide synthase (iNOS) and cyclooxygenase-2 (COX-2) (He et al., 2013).

### Anti-inflammatory effects *via* the Janus tyrosine protein kinase/signal transduction and transcription activator-mediated signaling pathway

**FIGURE 1:**
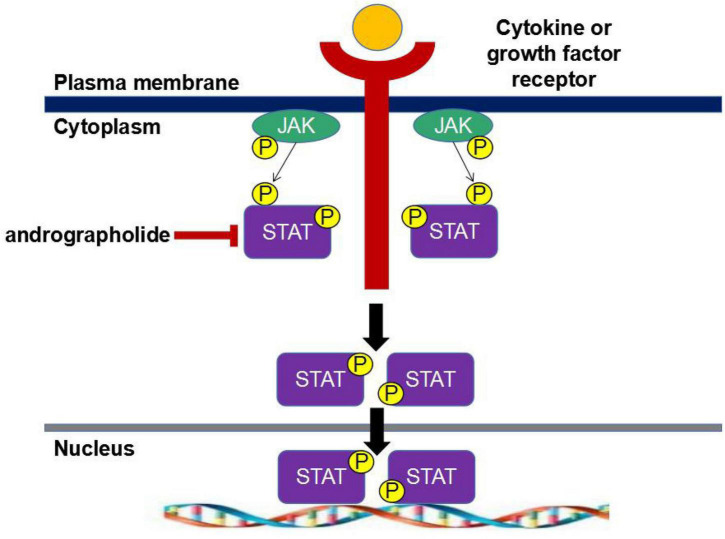
Janus tyrosine protein kinase/signal transduction and transcription activator-mediated signaling pathway.

Janus tyrosine protein kinase (JAK)/signal transducer and activator of transcriptional (STAT) signaling pathway is a commonly expressed intracellular signaling pathway, which is a common pathway for intracellular transmission of various cytokines. The STAT protein is one of the most conserved and effective transcription factors. To date, seven STAT proteins (STAT1, 2, 3, 4, 5A, 5B, and 6) have been identified to respond to tyrosine phosphorylation-activated transcription factors in response to cytokines and growth factors. Growth factors or cytokines bind to their homologous receptors, resulting in the dimerization of the receptors and the activation of receptor-associated Janus kinases (JAK1, JAK2, JAK3, or Tyk2), followed by the phosphorylation and conformational change of STAT protein through tyrosine, which is then transferred to the nucleus, binds to specific DNA, and regulates the transcription of thousands of pro-inflammatory cytokine genes (Figure 1 and Dodington et al., 2017). The chief role of the JAK/STAT-mediated signaling pathway is to regulate proliferation, differentiation, and apoptosis. It has been proved that andrographolide can exert anti-inflammatory effects by inhibiting the JAK/STAT signaling pathway. Andrographolide has been found to inhibit STAT1/2/3 phosphorylation and interfere with JAK/STAT signaling pathway in mice with influenza virus-induced inflammation by decreasing STAT1/2 phosphorylation (Figure 1; Chun et al., 2010; Ding et al., 2017). Parichatikanond et al. (2010) evaluated and compared the anti-inflammatory effects of diterpenoids extracted from *Andrographis*, including dehydro-andrographolide, AP1, andrographolide, AP2, and neoandrographolide, AP3, on inflammatory cytokine production and COX activity and found that AP2 significantly down-regulates the expression of JAK and STAT genes. In addition, AP2 and AP3 can significantly inhibit LPS-induced COX-2 activity, regulate the levels of LPS-induced pro-inflammatory cytokines such as TNF-α, IL-6, IL-1β, and IL-10 in human blood, act on various types of immune cells, and mediate inflammatory responses. In addition, activated COX is an important mediator of prostaglandin activation, and AP2 can down-regulate the expression of COX, thus preventing prostaglandin synthesis (Jiao et al., 2019). With an ovalbumin (OVA)-induced asthma inflammation model in mice, Peng et al. (2016) investigated the effects of andrographolide on the asthmatic condition. They discovered that andrographolide can reduce airway inflammation by regulating cytokines on helper T cells (Th17), inhibiting the expression of JAK1/STAT3 signaling pathways, and thus suppressing the asthmatic condition.

### Anti-inflammatory effects *via* T cell receptor-mediated signaling

Activation of cell receptors (TCR) promotes many signaling cascades that regulate cytokine production, cell proliferation, and differentiation to determine cell outcomes. Studies have shown that different concentrations of andrographolide can significantly reduce the levels of IFN-γ, IL-23, and IL-17 in patients with ulcerative colitis, thereby reducing the proportion of helper T cells Th1 and Th17. Liu et al. (2014) and Gao et al. (2020) both used a TNBS-induced mouse colitis model and found that andrographolide sulfonate not only reduced the mRNA and protein levels of pro-inflammatory cytokines but also improved colitis by inhibiting Th1/Th17 reaction. It improved intestinal epithelial injury and fibrosis caused by TNBS, confirming that andrographolide can be used for the treatment of gastrointestinal inflammatory diseases. It can be seen that the overaction of acquired immune cells (including Th1 and Th17 cells) is usually associated with intestinal inflammation (especially in IBD). From this vantage point, simultaneous targeting of Th1 and Th17 may be a viable strategy for regulating IBD, and the positive role of andrographolide in this context merits further investigation and discussion.

## Anti-tumor effect

**FIGURE 2:**
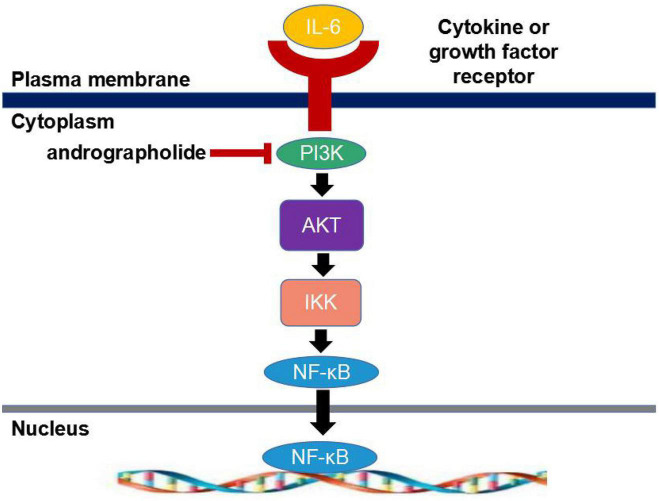
AKT-mediated signaling pathway.

Andrographolide is one of the principal antitumor components of *Andrographis*, which involves the activation, expression, and regulation of multiple genes. Andrographolide inhibits the growth of human lung cancer cells, human colon cancer cells, human osteosarcoma cells, and other tumor cells by inhibiting cell proliferation, inducing apoptosis in tumor cells, and blocking the cell cycle. Andrographolide can inhibit tumor activity: It was found that andrographolide inhibited the expression of Mir-21-5p and further promoted the expression of PDCD4 by inhibiting the NF-κB signaling pathway in a mmTV-PYMT breast cancer metastatic transgenic tumor mouse model to inhibit the proliferation, migration, and invasion of McF-7 breast cancer cells *in vitro* (Li et al., 2021). In conclusion, andrographolide not only mediates inflammatory responses through the NF-κB signaling pathway but also helps inhibit the growth of tumor cells through this pathway. Andrographolide inhibits the growth of colon cancer SW-480 cells by inhibiting Notch signaling. ROS production causes the arrest of SW-480 cells in the G0/G1 phase of the cell cycle. The expression of the pro-apoptotic protein Bax is upregulated, while the expression of the anti-apoptotic gene B-cell lymphoma-2 is downregulated (Bcl-2) (Khan et al., 2021). Andrographolide can also inhibit the growth, migration, and apoptosis of human glioma u87-MG cells by up-regulating the expression of apoptosis-related proteins caspase-3, Bax, and PARP and down-regulating the expression of anti-apoptotic protein Bcl-2 (Huang et al., 2020), thereby exerting its anti-glioma activity. Furthermore, inhibiting the PI3K/AKT pathway can inhibit human hepatoma cell proliferation and induce apoptosis (Fan et al., 2020). The PI3K/AKT signaling pathway is of vital importance in the body, with protein kinase B (AKT) as the core and phosphatidylinositol 3-kinase (PI3K) as the most important signal molecule upstream of the pathway. There are several important pathways downstream of AKT (including NF-κB) that regulate downstream signaling molecules (Figure 2).

## Antibacterial and antiviral

Studies indicate that andrographolide can interfere with the metabolism of amino acids and glucose in *Staphylococcus aureus*, reducing its pathogenicity by increasing bacterial nutrient intake to the environment. Since an increased glucose level can inhibit the toxicity of pathogens, andrographolide can indirectly play a role in decreasing bacterial virulence (Jin et al., 2019). From an antiviral standpoint, andrographolide can inhibit endoplasmic reticulum stress and cell apoptosis induced by the Chikungunya virus (CHIKV). The expressions of caspase-1, caspase-3, PARP, and IL-1β, IL-6, and interferon-gamma (IFN-γ) were down-regulated (Gupta et al., 2020b).

## Nervous system protection

Studies of andrographolide on the nervous system have been frequently reported in recent years. According to pharmacokinetic studies, andrographolide can be found in the heart, lungs, plasma, and brain (Bera et al., 2014). It is well known that brain tissue damage can lead to serious neurobehavioral disorders and promote severe inflammatory responses (Urday et al., 2015). Studies have shown that andrographolide derivative CX-10 can improve ischemic stroke (Yang et al., 2019). It was confirmed that CX-10 can effectively reduce the expression of TLR-4 and NF-κB, suggesting that inhibition of the TLR-4/NF-κB signaling pathway may be a potential mechanism underlying the neuroprotective effect of the andrographolide derivative CX-10. Yang et al. (2017) also reviewed the effects of andrographolide on brain injury in ischemic stroke. Andrographolide has also been shown to reduce both acute and secondary brain injury caused by cerebral hemorrhage (Li T. et al., 2018, Li X. et al., 2018). The phosphorylation and IκBα levels of P65 were decreased and nuclear translocation of P65 was inhibited by andrographolide, suggesting that this effect may be achieved by inhibiting NF-κB activation induced by intracerebral hemorrhage. We discovered that andrographolide, by acting on the NF-κB signaling pathway, not only exerts anti-inflammatory and anti-tumor effects but also has a protective role in the nervous system. In addition to the classic NF-κB signaling pathway, andrographolide can also mediate the PI3K/AKT signaling pathway to inhibit oxidative brain injury, and andrographolide can inhibit the expression of NOX2 and iNOS by inhibiting the PI3K/AKT signaling pathway (Chern et al., 2011). Diseases such as cerebral hemorrhage or injury can cause damage to endothelial cells of the brain and lead to thrombosis (Wang et al., 2016). It is important to reduce blood clots, which reduce blood flow to the brain and the survival of neurons. Studies have shown that andrographolide can reduce platelet aggregation (Thisoda et al., 2006), so andrographolide may interfere with thrombosis by reducing platelet activation and aggregation. Wang et al. (2019, 2020) observed the effects of andrographolide on hippocampal neuronal injury and cognitive function induced by chronic cerebral hypoperfusion (CCH) and found that andrographolide treatment can reduce the expression of TNF-α, IL-1β, caspase-3, and other inflammatory mediators and enhance the expression of BDNF and tyrosine kinase B (TrkB). CCH reversed hippocampal neuron damage and cognitive dysfunction in rats, and this may be related to the homologous loss of the phosphatase-tensin (PTEN)/AKT signaling pathway on human chromosome 10, which in turn involves an important AKT signaling molecule.

In terms of psychiatric diseases, andrographolide has antidepressant effects in addition to the previously mentioned improvement in Parkinson’s disease. It can improve the mental state of depressed mice that have been subjected to a variety of unpredictable stressors for an extended period of time by promoting the hippocampus brain-derived neurotrophic factor BDNF signaling pathway (Geng et al., 2019; Zhang et al., 2019). A large number of previous studies have found that the BDNF/TrkB signaling pathway is associated with depression, anxiety, and other related behaviors, as well as learning and memory (Soliman et al., 2010; Gibney et al., 2013). After BDNF is released, it binds to and activates TrkB, which ultimately mediates cell survival and synaptic regulation. Alzheimer’s disease (AD) is a degenerative disease, and andrographolide has been proved to have an obvious therapeutic effect on the AD model. Octodon degus can develop Alzheimer’s-like symptoms as they age, making them natural models of the disease (Braidy et al., 2012). Rivera et al. (2016) used a model of octodon degus and found that the cognitive function of octodon degus was improved after treatment with andrographolide, but this article did not involve the study of signaling pathways. According to our previous description, andrographolide can act on several classical pathways including NF-κB and AKT, which points to the direction of our research. In addition, andrographolide inhibits GSK-3β activity, which is involved in the Wnt signaling pathway (Tapia-Rojas et al., 2015). The association between the Wnt/β-catenin signaling pathway, hippocampal neurogenesis, and antidepressants has been found (Wta et al., 2022). These findings point us in a new direction as we continue to investigate psychiatric disorders *via* the Wnt/β-catenin signaling pathway.

The brain–gut axis has become a popular topic in recent years. In mice, andrographolide has been shown to disrupt the composition of intestinal flora (Wu et al., 2021). Gulf War syndrome (GWI) is a chronic, multi-symptom disorder characterized by neurological symptoms such as depression and pre-war anxiety (Blanchard et al., 2006). Saha et al. (2021) used andrographolide gavage to improve the intestinal microflora structure of GWI mice, significantly increasing the number of probiotics Akkermansia, Lachnospiraceae, and Bifidobacteria. Andrographolide also significantly restores IL-6 and Claudin-5 levels and increases the expression of the brain-derived neurotrophic factor BDNF. This suggests that further research on andrographolide is of great significance for the improvement of brain–gut axis-related diseases. Andrographolide research on the prevention and treatment of metabolic syndrome is increasing (Islam, 2017). Diabetes is well known to be associated with cognitive dysfunction, and diabetes is a recognized risk factor for AD (Biessels et al., 2006; Butterfield et al., 2014). Andrographolide has been shown to improve cognitive deficits in diabetic rat models, most likely due to increased acetylcholinesterase expression activity in the brain, which is inhibited by andrographolide in brain tissues (Thakur et al., 2016). In addition to diabetes, andrographolide also showed significant improvement in other metabolic diseases, such as obesity. Obesity is one of the risk factors for dementia (Gudala et al., 2013). Studies have demonstrated that andrographolide can prevent obesity in high-fat diet mice by improving lipid metabolism (Ding et al., 2014). Can andrographolide intervene in other metabolic diseases as it does in diabetes through the brain-gut axis? This is worthy of further investigation.

## Conclusion

In conclusion, the pharmacological effects of andrographolide include anti-inflammatory, antiviral, and antitumor properties, nervous system and liver protection, and other aspects, reflecting the efficacy and indications of andrographolide in Traditional Chinese Medicine for clearing heat and detoxifying, cooling blood, and detumescent. Furthermore, andrographolide also has pharmacological effects such as cardiovascular system protection and immune regulation, and its primary mechanism of action is closely related to anti-inflammatory and antioxidant properties. From the aforementioned andrographolide anti-inflammatory mechanisms, whether through JAK/STAT/NF-κB-mediated signaling pathways or the T cell receptor-mediated signal, andrographolide reflects an obvious role to achieve anti-inflammatory effects. However, the target of andrographolide’s direct mechanism of action has not been fully explained, and more experimental exploration is required. At present, the research on *Andrographis* has expanded from anti-inflammatory, antioxidant, and other mechanisms to clinical anti-tumor, treatment of heart diseases, and mental diseases, showing a very broad application prospect. In the next step, we should investigate the mechanism of action of andrographolide on depression in order for it to play a larger role in mental disorders.

## Author contributions

QC was actively engaged in writing. All authors participated in the manuscript’s review and editing.
